# Positive regulation of TNFR1 signaling via SH3 recognition motif

**DOI:** 10.3906/biy-2010-28

**Published:** 2021-04-20

**Authors:** Fatma Ece ÇOPUROĞLU, Fatma Zehra HAPİL, Şükran Burçak YOLDAŞ, Osman Nidai ÖZEŞ

**Affiliations:** 1 Department of Medical Biology and Genetics, Institute of Health Sciences, Akdeniz University, Antalya Turkey2; 2 ALTAY Therapeutics, San Francisco, CA USA

**Keywords:** TNF-α, TNFR1, Grb2, ERK, AKT

## Abstract

TNF is a pleiotropic cytokine and shows its biological function by binding to its receptors called TNFR1 and TNFR2. While TNFR1 induces apoptosis by activation of caspase-8 via the “death domain”, it also activates IKKα/β, MKK3/6, MKK4/7 by activation of TAK1. Although the TNFR1 signaling pathway is known by in large, it is not known how AKT and MAPKs p38, ERK1/2, and JNK1/2 are activated. The presence of a proline-rich PPAP region, (P448PAP451, a binding site for the SH3 domain-containing proteins) very close to the C-terminus promoted us to determine whether this region has any role in the TNFR1 signal transduction. To test this, the codons of P448 and P451 were changed to that of Alanin, GCG, via site-directed mutagenesis, and this plasmid was named as TNFR1-SH3-P/A. Subsequently, ectopically expressed the wild type TNFR1 and TNFR1-SH3-P/A in 293T cells and determined the levels of TNF-α-mediated phosphorylations of ERK, p38, JNK and AKT, NF-kB, and caspase-8 activation. While ectopic expression of our mutant diminished TNFα-mediated phosphorylations of p38, JNK, ERK and AKT, it increased NF-kB, and caspase-8 activations. In conclusion, TNFα-mediated ERK, AKT, JNK, p38 activations are affected by TNFR1 SH3 domain modifications.

## 1. Introduction

Tumor necrosis factor-alpha (TNFα) is a multifunctional cytokine. It has a detrimental role in the activation of the immune system, inflammation, differentiation and tumor toxicity (Sedger et al., 2014; Kalliolias and Ivashkiv, 2016). Two different types of signaling cascades are initiated after binding of TNFα to its receptors, TNFR1 and TNFR2 (Grel et al., 1994). TNFα binds to homotrimers of TNFR1. This facilitates binding of TRADD (tumor necrosis factor receptor type 1-associated death domain) to TNFR1 which then initiates the assembly of complex-1. The formation of complex-1 is known to be responsible for TNF-mediated activation of p38, JNKs and NF-kB. Therefore, activation of this promotes apoptosis (Micheau and Tschopp, 2003), necroptosis (He et al., 2009) and the activation of NF-kB (Boone E et al., 1998; Ozes et al., 1999; Fan et al., 2010). Upon the binding of TNFα, TNFR1 is activated and binds to DD (death domain) (Tartaglia et al., 1993) bearing proteins such as TRADD (TNF receptor-associated death domain) and RIP1 (receptor-interacting serine-threonine kinase 1) (Hsu et al., 1995; Hsu et al., 1996a, 1996b) via its DD located between the amino acids 356-441. The binding of TRADD and RIP1 to TNFR1 initiates the formation of complex-1, composed of the Fas-associated death domain (FADD) and procaspase-8, TRAF2 (TNF-receptor associated factor-2), LUBAC (linear ubiquitin chain assembly complex), cIAP1/2 (cellular inhibitor of apoptosis protein 1/2), and TAK1 (transforming growth factor-activated kinase-1) (Rothe et al., 1995; Shu et al., 1996; Park et al., 2004; Ea et al., 2006; Zheng et al., 2006; Jackson-Bernitsas et al., 2007; Bertrand et al., 2008; Varfolomeev et al., 2008; Haas et al., 2009; Tokunaga et al., 2009; Gerlach et al., 2011). In this complex, TAK1 is activated first, then it directly phosphorylates the mitogen-activated protein kinase kinases, MKK3/6, MKK4/7 (mitogen-activated protein kinase kinases) as well as IKK (inhibitor of nuclear factor-kB kinase) complex. The phosphorylations of these enzymes result in the activation of p38, JNKs (c-Jun N-terminal kinases), and NF-kB (nuclear factor-kappa B) pathway (Wang et al., 2001).

Even though the formation of complex-1 appears to explain how TNFα induces the activation of p38, JNKs, and NF-kB, it does not explain TNFα-mediated phosphorylations (activations) of the ERK (extracellular signal-regulated kinase), AKT (protein kinase B), and Stat3 (signal transducer and activator of transcription 3).

With this in mind, and knowing that phosphorylations of ERK1/2 and AKT require the activation of Grb2-Ras-RAF pathway (Zou J et al., 2019; Steelman et al., 2011), we postulated that the presence of SH3-domain (Src homology 3 domain) binding motif P448PAP451 at the C-terminal of TNFR1 could be a binding site for adaptor protein Grb2 (growth factor receptor-bound protein 2), which contains the SH3 domain. To test our hypothesis, we converted the codons of P448 and P451 to that of Alanine using site-directed mutagenesis on the TNFR1 expression vector. Transient transfection of this mutant vector into 293T cells resulted in ectopic expression of mutant TNFR1 protein. Then, we tested the impact of this mutant receptor on TNFα-mediated phosphorylation of p38, ERK1/2, AKT, and activations of NF-kB and caspase-8. In this study we show for the first time that the disruption of SH3 domain-binding motif of TNFR1 resulted in a diminishment of TNFα-induced phosphorylations of p38, ERK, AKT, and increased activation of NF-kB.

## 2. Materials and methods

### 2.1. Antibodies and reagents

Recombinant human TNFα was purchased from Sigma- Aldrich (St. Louis, MO, USA) (H8916), antibodies for TNFR1 (sc-8436), pERK (sc-7383), ERK (sc-94), pJNK (12882), JNK (7345), and GAPDH (sc-47724) were from Santa Cruz Biotechnology (Dallas, TX, USA). Monoclonal primary antibodies for pAkt (9271S), Akt (9272S) pp38 (9211L), and p38 (9212S) were from Cell Signaling Technology (Danvers, MA, USA) (NEB) and HRP conjugated secondary antibodies to mouse and rabbit IgG was from KPL (Gaithersburg, MD, USA) (474-1806 and 474-1506).

### 2.2. Plasmids and mutagenesis

TNFR1 expression plasmid was obtained by cloning TNFR1 ORF into pcDNA3.1A backbone. Following validation of vector efficiency by transfection into HEK293T cells, we proceeded with site direct mutagenesis. Site-directed mutagenesis was performed by Pfu polymerase (Agilent/Stratagene, La Jolla, CA, USA) reaction followed by Dpn1 digestion and plasmid sequences were verified by Sanger sequencing using Applied Biosystems 3130XL (Thermo Fisher Scientific, Waltham, MA, USA). The mutagenesis primers were: Forward: ‘5-cccgccgccctcgcgcccgcggccagtcttctcagatg**-**3’

Reverse: ‘5-catctgagaagactg**g**ccgcgggc**g**cgagggcggcggg-3’

### 2.3. Cell culture, transfections and treatments

HEK293T cells with passage numbers smaller than 22, were maintained grown in high glucose DMEM medium with stable L-glutamine (Lonza, Verviers, Belgium), supplemented with 10% fetal bovine serum (Gibco, Thermo Fisher Scientific) and 1% penicillin/streptomycin/amphotericin B (Biological Industries, Beit-Haemek, Israel), under standard 80%–90% humidity, 5% CO2 cell culture environment. CaPO4 precipitation method was utilized for transfections. TNFα stimulations were performed following 16 h serum starvation and 1-h pretreatment with 100 µM sodium orthovanadate (Na3VO4). 

### 2.4. Western blot

The cell lysate protein concentrations acquired in the lysis buffer [1.2% Triton X-100, 150 mM NaCl, 20 mM Tris-HCl (pH 7.4), 1 mM EGTA, 1 mM EDTA, 1 mM PMSF, 10 g/mL leupeptin, 0.15 U/mL aprotinin, 10 g/mL pepstatin A, and 1 mM Na3VO4] were calculated utilizing the Bradford method. One hundred micrograms of protein lysates were fractionated by SDS-PAGE on 10% polyacrylamide gels and transferred overnight to Immobilion-P PVDF membranes. Blots were probed with primary antibodies and corresponding HRP-conjugated secondary antibodies, and proteins were detected using Clarity ECL Western blotting substrate (1705061, Bio-Rad Laboratories, Hercules, CA, USA). 

### 2.5. Caspase-8 activation assay

Caspase-8 activities were measured according to the producer’s guidelines with a colorimetric caspase assay kit (K113-200, BioVision Inc. Milpitas, CA, USA). Concisely, 293T cells were treated or untreated for 24 h with 10 ng/mL TNF alpha at the 48th hour of transfection. Cells were lysed with cellular lysis buffer and protein concentration was determined. One hundred fifty micrograms lysate volume with cell lysis buffer was added to 50 μL and 50 μL 2X reaction buffer with 10 mM DTT was added. Lysates were incubated for 2 h with DEVD-pNA pNA (200 µM) and colorimetric measurements were made at a wavelength of 405 nm (nanometer).

### 2.6. NF-kB activation assay

The effect of mutant and wild type TNFR1 on NF-kB activity induced by TNFα was tested with the NF-Luc assay method. Concisely, 293T cells were cotransfected with plasmid reporter 150 ng/well (nanogram/well) NF-kB luciferase and 150 ng/well TNFR1 expression vectors by reverse transfection of Lipofectamine 2000. At transfection time of 48th hour, cells were incubated for 6 h with 10 ng/mL TNFα. Luciferase activity was assessed utilizing ONE-Glo luciferase assay system (E6120, Promega, Madison, WI, USA) as directed by the manufacturer. Briefly clarified, medium from the wells were changed and, 50 μL fresh medium was added. Fifty microliters ONE-Glo reagent was then applied to the wells and enabled for 3 min of lysis. Samples were moved quickly to luminometer plates and luminometric observations were performed. We normalized luciferase observations with MTT analysis to exclude cell number-caused variations.

### 2.7. Statistical analysis

The sensitivity of the Western blot image was calculated by ImageJ program. Graphs have been determined using Prism 6 (GraphPad Software) and data are presented as mean values ± SEM. All data were statistically analyzed by the Mann–Whitney U test. 

## 3. Results

### 3.1. Overexpression of wild type TNFR1 and TNFR1-SH3-P/A in 293T cells

**Figure 1:**
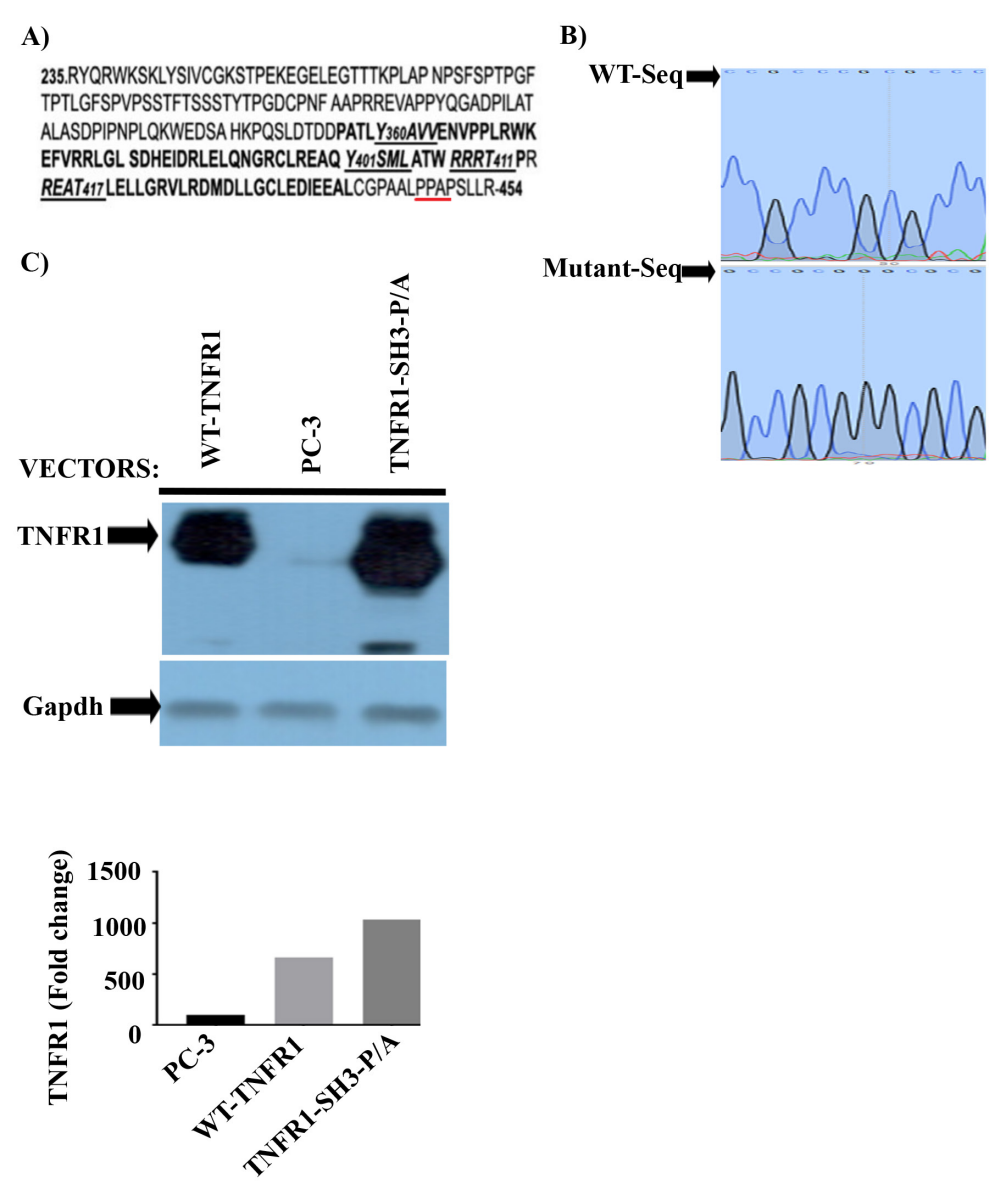
A proline-rich PPAP region, “P448PAP451”, a binding site for proteins containing the SH3 domain very close to the Cterminus, was discovered in the translation map of the TNFR1. The SH3 domain recognition sequence PPAP of TNFR1 is underlined in red. (A) The mutant TNFR1 was generated using site-directed mutagenesis. The Sanger sequence analysis was performed via Sanger sequencing, and the sequences of antisense strand of wild type and reverse strand of mutant TNFR1 were confirmed. (B) The wild type and mutant TNFR1 (TNFR1-SH3-P/A) can be ectopically expressed in 293T cells. (C) To determine ectopic expression of wild type and mutant TNFR1, both vectors were transfected into 293T cells (30 μg/10 cm plate) via calcium phosphate protocol for 48 h 100 μg of total cell lysates were used for western blotting. Fold changes of wild type and mutant proteins were determined by normalizing GAPDHcorrected values to that of background TNFR1.

As mentioned above, we have created SH3-binding domain mutant of TNFR1 by changing the codons of P448 and P451 to that of Alanine (Figure 1A) and confirmed the changes by Sanger sequencing (Figure 1B). Then, we demonstrated whether mutant TNFR1-SH3-PA protein can be expressed in 293T cells. To determine the ectopic expression of wild type and mutant TNFR1, 30 μg of plasmids were transfected into 293T cells using Ca-phosphate for 16 h, followed by medium replacement. Cell lysates were collected after 72 h of transfection and total cellular lysates were prepared in lysis buffer. One hundred micrograms of lysates were fractionated on 10% SDS-PAGE. As shown in Figure 1C, 293T cells express a very low level of endogenous TNFR1. However, after transfection, we achieved 600-fold wild type TNFR1 and 1000-fold TNFR1-SH3-P/A ectopic expression when normalized to background TNFR1 expression. 

### 3.2. The effect of TNFR1-SH3-P/A on TNFα-mediated ERK and AKT phosphorylations 

**Figure 2:**
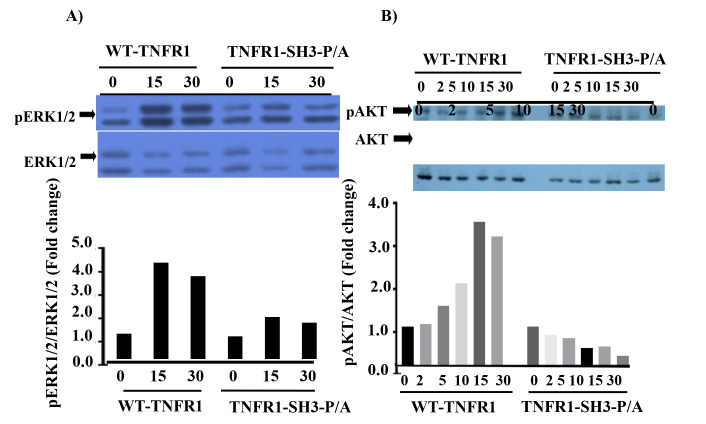
The mutant TNFR1 (TNFR1-SH3-P/A) interferes with TNFα-mediated phosphorylations of ERK1/2 and AKT kinases. Pattern of TNF-α-induced phosphorylations ERK1/2 (A) and AKT (B) were explored by western blot of 100 μg lysates obtained from 293T cells treated with TNF-α for the indicated time points. To determine the fold changes in phosphorylations, the band intensities of the images were determined using ImageJ, and the numerical values of phospho bands were divided into that of nonphospho bands.

We thought that TNFα-mediated phosphorylations of ERK1/2 and AKT simply cannot be explained by the formation of complex-1 during “canonical TNFα signaling”. To shed light on this issue, we considered that SH3-binding sequence PPAP at the far C-terminal of the TNFR1 could play a role in TNFα-induced activations of ERK1/2 and AKT, whose activations require Grb2-Ras-Raf pathway. To test this, we transiently transfected 293T cells with wild type and mutant plasmid for 2 days, serum-starved them for 16 h, then treated with TNFα (10 ng/mL). Since we previously showed the maximum time points of TNFα-induced phosphorylations of ERK1/2 and AKT (Hapil et al., 2020), we treated cells for 15 and 30 min to detect pERK1/2 and 2–30 min to detect pAKT. As shown in Figure 2A, TNFα induced 4.2-fold induction of ERK1/2 phosphorylation at 15 min and 3.8-fold induction of AKT phosphorylation at 30 min (Figure 2B) post-TNFα stimulation in wild type TNFR1 expressing cells. However, the induction of phosphorylation of ERK1/2 was only 1.7-fold in TNFR1-SH3-P/A expressing cells at 15th min post-TNFα stimulation (Figure 2A). More importantly, in TNFR1-SH3-P/A expressing cells TNFα-induced phosphorylation of AKT was completely ablated, in fact, background level of AKT phosphorylation gradually declined with time and at 30th min we detected only 1/5 of background level of AKT phosphorylation (Figure 2B). These results clearly indicate that SH3-binding domain of TNFR1 plays a significant role in transmission of signals which culminate in phosphorylations of ERK1/2 and AKT after TNFα stimulation. 

### 3.3. The effect of TNFR1-SH3-P/A on TNFα-mediated p38 and JNK1/2 phosphorylations

**Figure 3:**
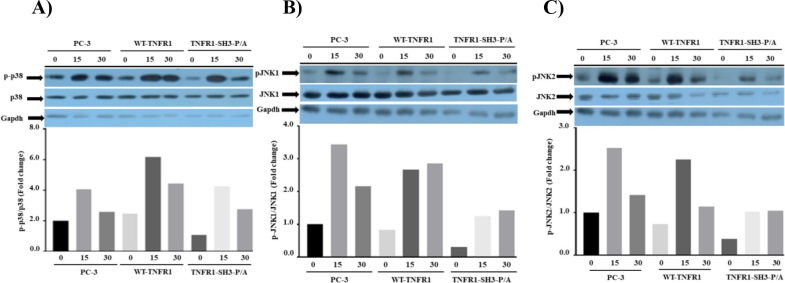
The mutant TNFR1 (TNFR1-SH3-P/A) interferes with TNFα-mediated phosphorylations of p38 and JNK kinases. Pattern of TNF-α-induced phosphorylations p38 (A) and JNK1 (B) and JNK2 (C) were explored by Western blot of 100 μg lysates obtained from 293T cells treated with TNF-α for the indicated time points. To determine the fold changes in phosphorylations, the band intensities of the images were determined using ImageJ, and the numerical values of phospho bands were divided into that of nonphospho bands.

According to “canonical TNFα signaling” formation of complex-1 and activation of TAK1 kinase in this complex is a must to phosphorylate and activate MKK3/6 and MKK4/7, which phosphorylate and activate p38 and JNK1/2, respectively. From this point of view, there seems to be no involvement of SH3-binding domain in activation of TAK1. However, after showing the effect of TNFR1-SH3-P/A mutant on phosphorylation of ERK1/2 MAPK, we wanted to test whether our mutant would affect TNFα-induced phosphorylations of the other MAPKs p38 and JNK1/2. Similar to ERK1/2 phosphorylation, we previously showed (Hapil et al., 2020) the 15th min as the maximum time point for TNFα-induced phosphorylations of p38 and JNK1/2. Therefore, in this experiment, we determined the level of phosphorylations of p38 and JNK1/2 at 15 and 30 min post-TNFα stimulation. As shown in Figure 3A, TNFα induced 6.3-fold induction of p38 phosphorylation at 15 min and this dropped to 4.2-fold at 30th min. In wild type-TNFR1 expressing cells, however, in 293T cells expressing mutant TNFR1 maximum level of phosphorylation was 4-fold at 15th min and this dropped to 2-fold at 30th min post-TNFα stimulation. When we look at the levels of phospho-JNK1 and 2 in the same lysates, we found 2.7- and 2.3-fold induction of phosphorylations of JNK1 and 2 at 15th min. post-TNFα stimulation in wild type TNFR1 expressing cells, respectively, Figures 3B and 3C. More importantly, phosphorylations of JNK1 and 2 in mutant TNFR1 expressing cells were only 1.2- and 1-fold at 30th min post-TNFα stimulation (Figures 3B and 3C). These results clearly indicate that PPAP sequence is somehow involved in the formation of complex-1 and activation of TAK1 after TNFα stimulation. 

### 3.4. The effect of TNFR1-SH3-P/A on NF-kB and caspase-8 activation 

The TNFα signaling resulting in activations of kinases ERKs, JNKs, p38s, AKTs, and NF-kB is very transient and is terminated by deubiquitinases A20 and CYLD (ubiquitin carboxyl-terminal hydrolase), which deubiquitinate TRADD, RIP1, NEMO (NF-kappa B essential modulator), and TRAF2. After the deubiquitination, the complex-1 disassembles and released TRADD/RIP1 translocates to the cytoplasm and participates in the formation of complex-2a which induces activation of caspase-8 and induction of apoptosis (Opipari et al., 1990; Trompouki et al., 2003; Malynn and Ma, 2009; Verhelst et al., 2014; Legarda et al., 2016).

**Figure 4:**
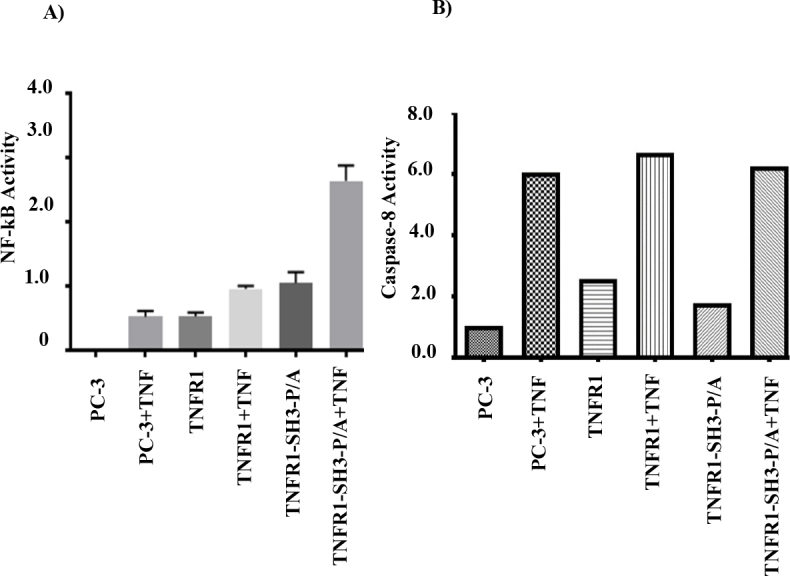
The mutant TNFR1 (TNFR1-SH3-P/A) augments TNFα-mediated induction of NF-kB, but does not affect caspase-8 activation. TNFR1-SH3-P/A mutant differentially affects TNFα-induced NF-kB activation. (A) To determine the NF-kB activity, 293T cells were transfected with 150 ng of NF-kB luciferase reporter plasmid along with 150 ng of PC-3, TNFR1, or TNFR1-SH3-P/A for 48 h, then cells were treated with 10 ng/mL TNFα for 6 h and luciferase activity was determined using Luminometer (Thermo Fisher Scientific). TNFR1-SH3-P/A mutant differentially affects TNFα-induced caspase-8 activation. (B) To determine the impact of mutant TNFR1 on caspase 8 activation, 293T cells were transfected with 150 ng of PC-3, TNFR1, or TNFR1-SH3-P/A for 48 h, then cells were treated with 10 ng/mL TNFα for 24 h and caspase-8 activation was measured by colorimetric caspase-8 activation assay kit.

Even though the PPAP sequence has not been related to induction of NF-kB or activation of caspase-8 in the past, and we showed that transient expression of our mutant TNFR1 unexpectedly affected TNFα-induced phosphorylations of p38 and JNK1/2, (activation of which does not require Grb2-Ras pathway), we thought that our mutants might also affect TNFα-induced activations of NF-kB and caspase-8. To test this, PC-3 (mock), wild-type TNFR1, and mutant TNFR1 vectors were transfected into 293T cells for 48 h, then cells were treated with 10 ng/mL TNFα for 24 h. Cellular lysates were collected and caspase-8 activation was measured immediately by colorimetric caspase-8 activation assay. As shown in Figure 4A, transient expression of wild type and mutant TNFR1 significantly increased background level of caspase-8 activity and mutant TNFR1 did not show any negative effect against TNFα-induced caspase-8 activation. To determine the effect of our mutant on TNFα-induced NF-kB induction, 293T cells were cotransfected with 150 ng/well NF-kB luciferase reporter plasmid and 150 ng/well TNFR1 expression vectors by Lipofectamine 2000 reverse transfection in 96-well plates. At the 48th hour of transfection, cells were treated with 10 ng/mL TNF-α for 6 h. At the end of treatment, luciferase activity was measured using ONE-Glo luciferase assay system (Promega, E6120) according to the manufacturer’s instructions. As shown in Figure 4B, expressions of TNFR1 increased background level of luciferase activity and this was further induced with TNFα stimulation. Unexpectedly, however, TNFR1-SH3-P/A mutant not only boosted background level of luciferase expression more than TNFR1, it also augmented TNFα-induced luciferase expression 3 times more than wild type TNFR1.

## 4. Discussion

TNFR1 is the death domain bearing receptor for the pleiotropic cytokine TNFα. In the past 30 years, initiation and transduction of TNFα signaling have been extensively studied and the proteins involved in these signaling pathways have been identified. Overall, TNFα signaling initiated from TNFR1 is very complex and organized via orderly bindings of TRADD-RIP1-TRAF2-cIAP1/2 and LUBAC to TNFR1. After the formation of this complex on TNFR1, TRADD-RIP1-NEMO and TRAF2 are ubiquitinated and these ubiquitinations serve as docking sites for TAB2/3-TAK1 and NEMO-IKKα-IKKβ complexes, resulting in the formation of complex-1 (Rothe et al., 1995; Hsu et al., 1995; Hsu et al., 1996; Hsu et al., 1996; Shu et al., 1996; Park et al., 2004; Ea et al., 2006; Zheng et al., 2006; Jackson-Bernitsas et al., 2007; Varfolomeev et al., 2008; Bertrand et al., 2008; Haas et al., 2009; Tokunaga et al., 2009; Gerlach et al., 2011). In complex-1, TAK1 is activated by autophosphorylation and activated TAK1 phosphorylates and activates MKK3/6, MKK4/7, and IKKs. Activated IKKs induce NF-kB activation whereas MKK3/6 and MKK4/7 phosphorylate and activate p38s and JNKs, respectively. Overall, the ultimate target activated in complex-1 is TAK1, and TAK1 activation can explain how TNFα induces activations of NF-kB, JNK1/2, and p38 (Wang et al., 2001).

Even though TNFR1 does not bear the PI3Kp85-binding site, YXXM motif, we have previously shown that PI3Kp85 binds to TNFR1 and induces synthesis of phosphatidylinositol three phosphates (PIP3) by activating PI3K (phosphoinositide 3-kinase) pathway, which ultimately induces phosphorylation of AKT kinase (Ozes et al., 1999; Ozes et al., 2001; Pincheira et al., 2008). Since we cannot explain TNFα-mediated phosphorylation of AKT by the formation of complex-1, we decided to look at different domains of TNFR1 and found that TNFR1 has SH3-recognition sequence, PPAP, at the C-terminus of TNFR1, Figure 1A. This prompted us to think that mechanism of activation of PI3K may rely on PPAP motif and adaptor proteins bearing SH3 domain such as Grb2 can bind to this domain. Indeed, binding of Grb2 to TNFR1 has been shown before (Hildt and Oess, 1999), however, they did not perform any mechanistic studies. Having this in mind, we mutated PPAP sequence to APAA sequence by site-directed mutagenesis and showed the expression of mutant TNFR1 in 293T cells (Figures 1B and 1C). As we anticipated, the ectopic expression of mutant TNFR1 not only inhibited TNFα-mediated phosphorylations of ERK1/2, but also it completely inhibited TNFα-mediated phosphorylation of AKT kinase (Figures 2A and 2B). 

Downward’s team showed that the Ras-GTP complex not only binds to RAF (Proto-oncogene serine/threonine-protein kinase) but also binds to catalytic subunit p110α of PI3K. This binding activates PI3K complex which produces PIP3 from PIP2, and resulting PIP3 binds to PDK1 (phosphatidylinositol-activated kinase), activate this kinase and activated PDK1 phosphorylate and activate AKT. These breakthrough findings explained GRB2-RAS-RAF-MEK-ERK and GRB2-RAS-PI3K-PDK1-AKT pathways (Rodriguez-Viciana et al., 1994). This breakthrough findings of Downward’s team explained as to how GRB2-RAS-RAF-MEK-ERK as well as GRB2-RAS-PI3K-PDK1-AKT pathways. When we look at our results in Figures 2A and 2B, this is exactly what we see on TNFR1. The presence of Grb2-binding site is initiating the formation of both GRB2-RAS-RAF-MEK-ERK as well as GRB2-RAS-PI3K-PDK1-AKT complexes and mutation of PPAP to APAA significantly affect phosphorylation of ERKs and ablated TNFα-induced phosphorylation of AKT. Recently, we have shown that TNFR1 bears two tyrosine phosphorylation sites (Y360, Y401) and these sites are phosphorylated upon TNFα stimulation and these sites can initiate Grb2-Ras-RAF-ERK pathway. However, according to our results Grb2-RAS-p110α-complex seems to form primarily at PPAP site and initiates signaling pathways which result in activation of ERKs and AKT. 

As mentioned above, TNFR1-induced phosphorylations of p38 and JNK kinases are mediated by activation of TAK1 in the complex-1. However, it was recently shown that ASK1 can be recruited to TNFR1 by A20 in a complex-1-independent manner and ASK1 can phosphorylate JNK1/2 (Won et al., 2020). Also, Patel et al. have shown that ASK1 can phosphorylate p38 kinases (Patel et al., 2019). Since changing PPAP site to APAA dramatically affected TNF**α**-induced phosphorylation of p38/JNKs, it is possible that the structure of mutant TNFR1 cannot accommodate the binding of ASK1 to TNFR1 resulting in diminishment of TNFR1-induced phosphorylations of JNKs and p38 kinases.

**Figure 5:**
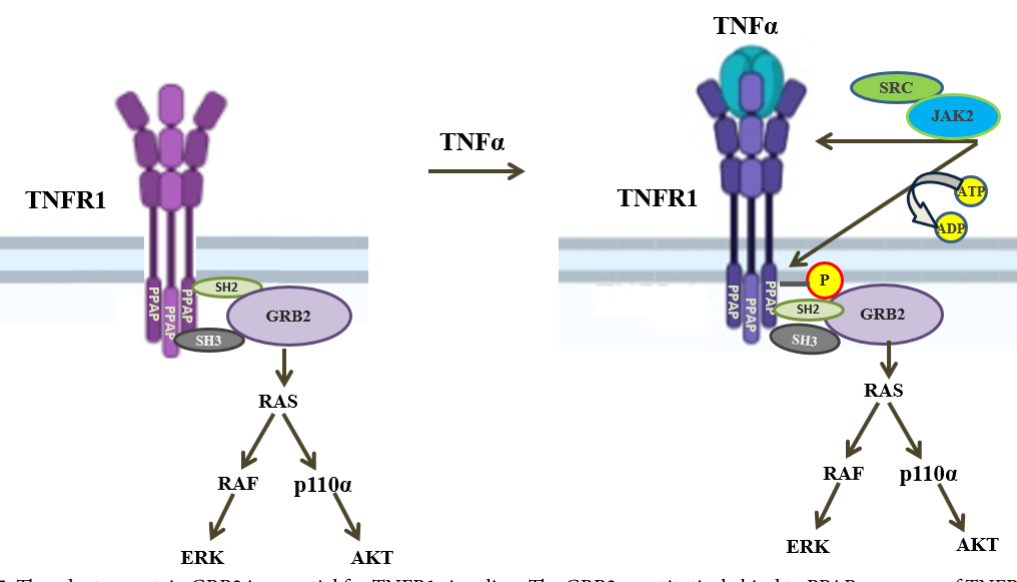
The adaptor protein GRB2 is essential for TNFR1 signaling. The GRB2 constitutively bind to PPAP sequence of TNFR1 via its SH3 domain. After TNFα binding to TNFR1, cSRC and JAK2 (which constitutively bind to TNFR1) phosphorylate Y360 and Y401. The phosphorylated tyrosines were represented as single (P) on TNFR1. GRB2 binds to these tyrosine phosphorylated amino acids via its SH2 domain. TNFR1-bound GRB2 binds to RAS and initiate RAS-induced RAS-RAF-ERK and RAS-p110α -AKT pathways.

Since Grb2 has both SH3 and SH2 domains (phosphotyrosine recognition domain), based on our results, we postulate that after binding to PPAP via SH3 domain its SH2 domain can interact with phosphorylated Y360/Y401. In fact, this is the most likely scenario of TNFR1 signaling. Since GRB2 constitutively binds to TNFR1 (Hapil et al., 2020), it can always initiate the activation of RAS-controlled RAF and p100a pathways. By doing so, the association of GRB2 with TNFR1 causes persistent generation of signals from TNFR1, and addition of TNF**α** can only further increase the activation of RAS-RAF and RAS-p110α pathways by simply increasing the GRB2 load on TNFR1. Indeed, unlike other cytokines and growth factors-mediated activation of ERK and AKT we studied, the level of TNF**α**-induced activations of ERK and AKT are always modest. The mode of action of GRB2 on TNFR1 signaling is illustrated in Figure 5. In conclusion, PPAP sequence of TNFR1 seems to have significant role in initiating signaling events resulting in activations of ERK, AKT, JNKs and p38 kinases by TNFα.
